# Structure-Activity Relationship Study of Sesquiterpene Lactones and Their Semi-Synthetic Amino Derivatives as Potential Antitrypanosomal Products

**DOI:** 10.3390/molecules19033523

**Published:** 2014-03-21

**Authors:** Stefanie Zimmermann, Gerda Fouché, Maria De Mieri, Yukiko Yoshimoto, Toyonobu Usuki, Rudzani Nthambeleni, Christopher J. Parkinson, Christiaan van der Westhuyzen, Marcel Kaiser, Matthias Hamburger, Michael Adams

**Affiliations:** 1 Department Pharmaceutical Biology, University of Basel, Klingelbergstrasse 50, 4056 Basel, Switzerland; E-Mails: stefanie.zimmermann03@gmail.com (S.Z.); maria.demieri@unibas.ch (M.D.M.); matthias.hamburger@unibas.ch (M.H.); 2 Department Medical Parasitology & Infection Biology, Swiss TPH, Socinstrasse 57, 4000 Basel, Switzerland; E-Mail: marcel.kaiser@unibas.ch; 3 CSIR Biosciences, Meiring Naudé Road, Brummeria, Pretoria 0001, Gauteng, South Africa; E-Mails: gfouche@csir.co.za (G.F.); rnthambeleni@csir.co.za (R.N.); cwvdwesthuyzen@csir.co.za (C.W.); 4 Department of Materials and Life Sciences, Faculty of Science and Technology, Sophia University, 7-1 Kioicho, Chiyoda, Tokyo 102-8554, Japan; E-Mails: tkmh-937@sophia.ac.jp (Y.Y.); t-usuki@sophia.ac.jp (T.U.); 5 School of Biomedical Sciences, Charles Sturt University, Orange, NSW 2800, Australia; E-Mail: cparkinson@csu.edu.au; 6 University of Basel, Petersplatz 1, 4003 Basel, Switzerland

**Keywords:** cynaropicrin, sesquiterpene lactones, antitrypanosomal, cytotoxicity, structure-activity-relationship, dimethylamino analogues, *T. b. rhodesiense* acute mouse model

## Abstract

Sesquiterpene lactones (STLs) are natural products that have potent antitrypanosomal activity *in vitro* and, in the case of cynaropicrin, also reduce parasitemia in the murine model of trypanosomiasis. To explore their structure-antitrypanosomal activity relationships, a set of 34 natural and semi-synthetic STLs and amino-STLs was tested *in vitro* against *T. b. rhodesiense* (which causes East African sleeping sickness) and mammalian cancer cells (rat bone myoblast L6 cells). It was found that the α-methylene-γ-lactone moiety is necessary for both antitrypanosomal effects and cytotoxicity. Antitrypanosomal selectivity is facilitated by 2-(hydroxymethyl)acrylate or 3,4-dihydroxy-2-methylenebutylate side chains, and by the presence of cyclopentenone rings. Semi-synthetic STL amines with morpholino and dimethylamino groups showed improved *in vitro* activity over the native STLs. The dimethylamino derivative of cynaropicrin was prepared and tested orally in the *T. b. rhodesiense* acute mouse model, where it showed reduced toxicity over cynaropicrin, but also lost antitrypanosomal activity.

## 1. Introduction

Sleeping sickness, or human African trypanosomiasis (HAT), is a deadly protozoal disease caused by *Trypanosoma brucei* species spread by tsetse flies (*Glossina spp.*). The two human pathogenic subspecies, *T. b. rhodesiense* (95% of cases) and *T. b. gambiense* (5% of cases), differ in terms of their geographic distribution, clinical pictures, and the drugs used to treat parasitemia [1]. Currently, there are about 30,000 new HAT cases annually, and as many as 30 million people live in HAT endemic areas [2]. Despite some recent successes like nifortimox-eflornithine combination therapy (NECT) [3], HAT drugs are still inefficient by modern standards and need to be replaced by compounds that are safer and easier to administer [4]. 

Natural products from plants have been instrumental in developing drugs to treat protozoal diseases, such as quinine and artemisinin against malaria [5,6]. Currently, though, no natural product-based antitrypanosomal drugs are in use or even in late stage clinical development. Recently, we reported the *in vivo* activity [7] and mode of action [8] of cynaropicrin (**1**), a natural sesquiterpene lactone (STL). Although more than 883 plant-derived small molecules have shown antiprotozoal (antitrypanosomal, antiplasmodial, and antileishmanial) effects *in vitro*, of which 87 were STLs [5,6], this was the first reported plant compound with *in vivo* anti-*T. brucei* action specifically. STLs are a chemotaxonomic feature of the largest plant family, the Asteraceae [9], and to date more than 5,000 STLs are known [5,10]. STLs are a promising compound class for antitrypanosomal drug discovery [11,12,13], thus a better understanding of the structural features that contribute to activity is expedient [14,15]. This study explores the antitrypanosomal structure-activity relationships within this compound class using a group of 18 molecules comprising natural STLs and 16 semi-synthetic STL-amines and two other derivatives against *T. b. rhodesiense in vitro*. Mammalian cancer cells (L6 cell line) were used to evaluate cytotoxicity. The antitrypanosomal effects of the eight compounds **1**, **2**, **5**–**9**, and **13** have been reported before [7,11,12], but were included for comparison. Additionally, based on the *in vivo* antitrypanosomal effects of **1** after intraperitoneal application [7,8], **1** and the dimethylamino derivative **19** were tested *in vivo* in the acute mouse model with oral application. The rationale behind derivative **19** was that masking the α,β-unsaturated enoate in the lactone ring would possibly create a prodrug with increased water solubility, improved pharmacokinetic properties, and reduced unspecific binding to biological thiols via Michael addition to the α-methylene-γ-lactone. Through subsequent bioactivation it would be converted to the parent compounds and, hence, display its biological activity on the target. A similar approach had been previously successfully applied to several STLs with anticancer activity like helenalin, costunolide, and parthenolide [16].

## 2. Results and Discussion

**Figure 1:**
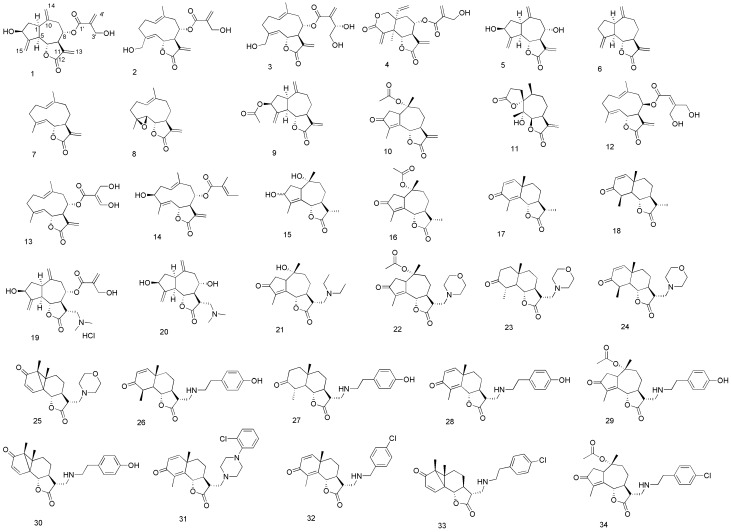
Structures of sesquiterpene lactones (STLs) **1**–**18** and semi-synthetic STL amino derivatives **19**–**34**.

The *in vitro* antitrypanosomal activity of compounds **1**–**34** was determined against *T. b. rhodesiense* (STIB 900 strain), while their cytotoxicity was evaluated using rat myoblast L6 cells to determine the selectivity indices (SI; IC_50_ L6/IC_50_ *T. b.*) of each compound (Figure 1, Table 1).

Cynaropicrin (**1**) and onopordopicrin (**2**), which both have 2-(hydroxymethyl)acrylate side chains, showed IC_50_s of 0.3 and 0.4 µM, and SIs of 7.8 and 8.2 [7,8,12], respectively. Extension of the side chain in **2** to a 3,4-dihydroxy-2-methylenebutylate, as in cnicin (**3**), had little effect on activity (0.4 µM) or selectivity (SI 10). Compound **5**, which lacked the 2-(hydroxymethyl)acrylate side chain of **1**, had a 16 fold lower antitrypanosomal activity (4.9 µM) and a nine fold lower cytotoxicity (19 µM) as compared to **1** [8]. Compound **14**, a 2-methylbut-2-enoate (tiglate) STL, was less active and selective than the 2-(hydroxymethyl)acrylate STLs **1**, **2**, and **3**. Compounds **15**–**18**, which lack an exocyclic methylene group in the lactone ring, showed reduced antitrypanosomal activities (IC_50_s > 12 μM) and a total lack of cytotoxicity. 

**Table 1:** *In vitro* activity of compounds against STIB 900 strain and L6 cells. Citations are shown for previously reported antitrypanosomal compounds.

Compound	*T. brucei* STIB 900 IC_50_ (μM) ^a^		L6 cells IC_50_ (μM) ^a^	SI ^b^
1 [7,8]	0.3 ± 0.001		2.2 ± 0.3	7.8
2 [12]	0.4 ± 0.01		3.1 ± 1.1	8.2
3	0.4 ± 0.1		4.2 ± 0.9	10
4	0.2 ± 0.01		0.6 ± 0.02	3
5 [8]	4.9 ± 0.34		19.2 ± 3.2	3.9
6 [11]	4.4 ± 1.2		8.3 ± 1.9	1.9
7 [11]	1.3 ± 0.4		7.7 ± 1.3	5.9
8 [11]	0.8 ± 0.5		5.2 ± 0.9	6.5
9 [11]	10.8 ^c^		15.6	1.4
10	0.6 ± 0.2		4.3 ± 0.5	7.6
11	5.8 ± 0.7		6.9 ±1.8	1.2
12	0.9 ± 0.2		2.2 ± 0.1	2.5
13 [11]	1.2 ± 0.2		1.6 ± 0.1	1.3
14	3.1 ± 0.3		10.5 ± 0.1	3.4
15	54.7 ± 8.0		353.2 ± 4.0	6.4
16	45.7 ± 5.0		>292.2	>6.4
17	41.5 ± 0.8		>365.9	>8.8
18	12.9 ± 2.4		34.0 ± 1.5	2.6
19	0.5 ± 0.003		5.2 ± 1.3	10.4
20	3.6 ± 1.0		8.6 ± 1.3	2.5
21	4.2 ± 0.8		9.4 ± 2.2	2.2
22	0.7 ± 0.1		7.4 ± 0.8	10.3
23	11.8 ± 2.7		65.6 ± 9.7	5.6
24	2.4 ± 0.7		31.3 ± 1.4	13.3
25	2.6 ± 0.5		9.9 ± 1.6	3.8
26	6.7 ± 1.3		>236.2	>35.0
27	13.0 ± 1.4		45.6 ± 3.2	4
28	13.4 ± 1.1		87.9 ± 2.2	6.5
29	6.6 ± 0.7		22.1 ± 4.0	3.3
30	9.9 ± 1.6		31.8 ± 1.3	3.2
31	7.0 ± 2.2		21.8 ± 2.7	3.1
32	10.6 ± 1.5		34.8 ± 1.2	3.4
33	10.2 ± 3.2		27.6 ± 6.7	2.7
34	5.4 ± 1.1		22.5 ± 1.6	4.2
Melarsoprol ^d^	0.01 ± 0.01		21.7 ± 8.7	2170
Podophyllotoxin ^e^			0.02 ± 0.01	

^a^ Average of three independent assays. ^b^ Selectivity Index (SI): IC_50_ against L6 cells divided by IC_50_ against STIB 900 strain. ^c^ tested once. ^d^ positive control for STIB 900 assay. ^e^ positive control for cytotoxicity assay.

The antitrypanosomal activity of the semi-synthetic dimethylamino derivative **19** (IC_50_ 0.5 µM) was moderately higher than that of the parent compound **1** (0.3 µM). Compound **21**, a diethylamine derivative of **16**, was markedly more active than its parent compound (IC_50_ 4.2 µM for **21**, compared to 46 µM for **16**). The morpholino STL derivatives **22**–**25** exhibited fairly wide variations in activity. The adduct **22** (IC_50_ 0.7 µM) was as potent as starting methylene compound **10** (IC_50 _0.7 µM), with slightly increased selectivity (SIs of 10.3 and 7.6, respectively). Compounds **23** and **24** showed low cytotoxicity (IC_50_s 65.6 µM and 31.3 µM, respectively), but **24** had much higher antitrypanosomal activity (IC_50_ 2.4 µM) than **23** (IC_50_ 11.8 μM). Compound **25** had a similar (IC_50_ 2.6 µM) activity against *T. b. rhodesiense* as **24**. 

The STL tyramine adducts 26–30 showed low cytotoxicities (IC_50_s 22.1 µM to > 236 µM), and 29 had the highest antitrypanosomal activity (IC_50_ 6.6 µM) of the Michael adducts tested. The 1-(2-chlorophenyl)piperazinyl STL 31 and the 2-(4-chlorophenyl)ethylamino STLs 32-34 all showed low antitrypanosomal activity (IC_50_s > 5 µM) and modest cytotoxicity (IC_50_s > 20 µM).

We recently reported *in vivo* antitrypanosomal effects of **1** [7,8]. Upon intraperitoneal administration, the parasitemia was decreased over several days, but the compound was not able to cure the mice when they were treated with 10 mg/kg/b.i.d. for four consecutive days. In an attempt to improve thebioavailability, the exocyclic double bond in the lactone ring was masked to obtain water soluble dimethylamino derivative **19**. Compounds **1** and **19** were orally administered in the acute sleeping sickness mouse model. Four mice, each treated with 50 mg/kg body weight/day of **1**, showed reduced parasitemia on day 7 after infection. However, the animals were euthanized on day 10 post infection due to obvious signs of cytotoxicity of the compound. Mice treated with compound **19** exhibited less signs of toxicity. However, the compound showed no *in vivo* efficacy, since the mean survival time was the same as for the control (Table 2). 

**Table 2:** Activity of compound **1** and **19** in the STIB 900 mouse model of trypanosomiasis.

Compound	RA ^a^	dose (mg/kg)	survival (days) ^b^
**1**	po	4 × 50	9.5
**19**	po	4 × 50	10
	po	4 × 25	8.5

^a^ RA, route of administration: per oral (po). ^b^ Average days of survival of all mice; untreated controls euthanized at day 10 post infection.

### Discussion

This structure-activity relationship (SAR) study showed the STL 2-(hydroxymethyl)acrylates **1**, **2**, and **4** along with the STL 3,4-dihydroxy-2-methylenebutylate **3** are the most active and selective STLs against *T. b. rhodesiense*. These compounds have two active α,β-unsaturated enoate moieties in common, one on the lactone ring and the other on the side chain. Compound **10** has no side chain with an enoate moiety, but does possess a cyclopentenone group, which can serve as the additional reactive enone. In fact, vernodalin (**4**), which has a third reactive α,β-unsaturated enoate group, had slightly higher potency (along with greater toxicity) than **1**. STLs lacking a reactive α,β-enoate function in their side chains (**12**, **13**, and **14**) or with no side chain (**5**, **6**) showed weaker antitrypanosomal and cytotoxicity effects compared to their analogues (**1**, **2**, and **3**). The results are in accordance with findings by others [16,17,18]. Schmidt *et al.* showed in a SAR study using 40 STLs that bifunctionality as Michael acceptors is very important for a high level of antitrypanosomal activity but that very similar structural features determine both antiprotozoal and cytotoxic activity [14]. This was recently confirmed by the same authors with a larger data set of over 70 STLs [17]. Many bioactivities in STLs have been attributed to a Michael addition of the methylene-γ-lactone moiety to biological thiols [19]. Recent findings on the adduct formation of the two α,β-unsaturated nucleophilic enoate groups at C13 and C3' in **1** with trypanothione and glutathione in trypanosomes corroborated the long presumed mode of action of STLs, in addition to an inhibition of ornithine decarboxylase [8]. The same mode of action can be expected for **2**, **3**, and **4**. Regarding the 14 semisynthetic STL amines tested here, it was observed that the addition of morpholino and dimethylamino groups maintained or even enhanced the *in vitro* activity compared to their parent structures, whereas tyramino-, 2-(4-chlorophenyl)ethylamino- and 1-(2-chlorophenyl)piperazyl derivatives were generally two to four fold more active than their parent compounds having no enoate moiety, albeit with the disadvantage of higher toxicity (SI in the range 3–7, generally). 

Compared to **1**, the toxicity and antitrypanosomal activity of **19** in the *T. brucei rhodesiense* acute mouse model after oral application was poorer. Since the amino derivative **19** –in spite of a reasonably high level of *in vitro* activity- did not show any *in vivo* efficacy, it will have to be investigated in further studies whether other types of derivatives could be useful prodrugs of STLs for the p.o. treatment of HAT. 

## 3. Experimental

### 3.1. Test Compounds

Cynaropicrin (**1**) was isolated from artichoke leaves as previously reported [7]. Compound **5** was prepared by mild alkaline hydrolysis of **1** as described by Zimmermann *et al*. [7]. Zaluzanin D (**9**) and dehydrocostuslactone (**6**) were isolated from *Saussurea costus* as referenced [11]. Compound **10** [20], the precursors for synthesis of compounds **23**‒**31**, and as well as the synthesis of **16** [20,21] were performed according to literature methods. Vernodalin (**4**) was provided from Prof. Hajime Ohigashi, Kyoto University, Japan. Psilostachyin A (**11**) was kindly supplied by Dr. Wolfgang Schühly, University of Graz, Austria. Onopordopicrin (**2**) was isolated from *Arctium nemorosum* [12]. Nobilin (**14**) was kindly supplied by Prof. Imanidis from the University Applied Sciences and Arts Northwestern Switzerland. Compound **12** was from Prof. Merfort, University of Freiburg, Germany. Cnicin (**3**) was isolated from *Cnidus benedictus* L [22]. Eupatoriopicrin (**13**), costunolide (**7**), and parthenolide (**8**)were isolated from *Saussurea costus* [11]. Santonin (**17**) was purchased from Fluka Chemie (Buchs, Switzerland, >98% purity). 

### 3.2. General Experimental Information

NMR spectra were run on a 400 MHz Varian INOVA instrument. Samples were referenced against chloroform at 77.00 ppm for ^13^C and against tetramethylsilane at 0.00 ppm for ^1^H. High resolution mass spectra were recorded on a Waters SYNAPT G1 HDMS mass spectrometer operated in electrospray mode. Leucine enkephalin (50 pg/mL) was used as reference calibrant to obtain typical mass accuracies between 1 and 3 mDa. Melting points were determined using a Mettler FP62 capillary melting point apparatus and are uncorrected. All reagents were of reagent grade purchased from Sigma-Aldrich (Schnelldorf, Germany) and were used without any further purification. Solvents used for chromatography or extractions were distilled prior to use. Thin-layer chromatography was carried out using pre-coated aluminum-backed plates (Merck Silica Gel 60 F_254_). Column chromatography was performed on Fluka silica gel 60 (70–230 mesh). Dry solvents were purified as described by Perrin and Armarego [23]. All starting materials were obtained commercially and used without further purification.

### 3.3. Syntheses and Spectral Data of Analogues **15**, **21**–**34**

*3-Hydroxyisophotosantonin* (**15**). A solution of *O*-acetylisophotosantonin (1.475 g, 4.813 mmol) in MeOH (49 mL) at 0 °C was treated with sodium borohydride (0.293 g, 7.750 mmol) carefully. The reaction was left at 0 °C for 3 h, then left to warm to room temperature overnight. The mixture was extracted from saturated aqueous NH_4_Cl (50 mL) with ethyl acetate (3 × 50 mL), the extracts pooled and dried (MgSO_4_). The dried filtrate was concentrated to a tacky white foam, then dissolved in absolute ethanol (16 mL). 5% Aqueous KOH (150 mL) was added and the mixture stirred for 18 h at room temperature. The mixture was acidified to pH < 2 with 18% aqueous HCl, stirred for 30 min, extracted with ethyl acetate (3 × 50 mL) and washed with saturated aqueous K_2_CO_3_. Concentration yielded a yellow solid, which was recrystallized (EtOAc/hexane) to a white amorphous powder (0.313 g, 36%). NMR showed an approximately 2.2:1 mixture of secondary alcohols had been isolated. *Major isomer*: ^1^H-NMR (400 MHz, CDCl_3_ + CD_3_OD): δ 4.73 (1H, d, *J =* 11.0 Hz, H-6), 4.49 (1H, t, *J =* 6.8 Hz, H-3), 2.90 (1H, ~ br td, *J =* 7.5 and 1.9 Hz, H-1), 2.45 (1H, dt, *J =* 13.8 and 8.0 Hz, H-2a), 2.34–2.12 (2H, m, H-9a and H-11), 2.03–1.91 (2H, m, H-7 and H-9b), 1.89 (3H, s, C-10 *Me*), 1.65 (1H, dd, *J =* 16.0 and 2.3 Hz, H-8a), 1.61 (1H, td, *J =* 13.7 and 6.7 Hz, H-2b), 1.46–1.30 (1H, m, H-8b), 1.22 (3H, d, *J =* 6.9 Hz, C-11 *Me*), 1.03 (3H, s, C-4 *Me*); ^13^C-NMR (101MHz, CDCl_3_ + CD_3_OD): δ 178.82 (C, C-12), 143.96 (C, C-14), 131.28 (C, C-5), 82.04 (CH, C-6), 76.97 (C, C-10), 74.40 (CH, C-3), 54.31 (CH, C-1), 48.92 (CH, C-7), 44.87 (CH_2_, C-9), 41.34 (CH, C-11), 34.36 (CH_2_, C-2), 25.41 (CH_2_, C-8), 20.88 (CH_3_, C-4 *Me*), 13.23 (CH_3_, C-11 *Me*), 12.23 (CH_3_, C-10 *Me*); *minor isomer*: ^1^H-NMR (400MHz, CDCl_3_ + CD_3_OD): δ 4.64 (1H, d, *J =* 11.0 Hz, H-6), 4.54 (1H, d, *J =* 7.1 Hz, H-3), 1.92 (3H, s, C-10 *Me*), 1.22 (3H, d, *J =* 6.9 Hz, C-11 *Me*), 0.91 (3H, s, C-4 *Me*); ^13^C-NMR (101 MHz, CDCl_3_ + CD_3_OD): δ 178.71 (C, C-12), 143.79 (C, C-4), 133.32 (C, C-5), 81.71 (CH, C-6), 79.59 (C, C-10), 74.23 (CH, C-3), 55.57 (CH, C-1), 48.96 (CH, C-7), 44.27 (CH_2_, C-9), 41.51 (CH, C-11), 34.81 (CH_2_, C-2), 25.26 (CH_2_, C-8), 20.56 (CH_3_, C-4 *Me*), 13.23 (CH_3_, C-11 *Me*), 12.20 (CH_3_, C-10 *Me*); HRMS (ESI) calculated for C_15_H_21_O_3_ 249.1491; found 249.1423 (MH^+^–H_2_O); and calculated for C_15_H_21_O_4_ 265.1440; found 265.1392 (MH^+^–H_2_).

*Cynaropicrin dimethylamine adduct hydrochloride* **19**. To a cold solution of **1** (0.50 g; 1.44 mmol) in absolute EtOH (15 mL), dimethylamine, (0.72 mL, 2.0 M solution in MeOH) was added under argon atmosphere. The solution was stirred at 5 °C for 5 h, then, after concentration of the solvent under reduced pressure, the crude compound was recrystallized from acetone/Et_2_O. The amino adduct (0.222 g; 0.566 mmol) was then dissolved in MeOH (5 mL) and a solution of HCl (0.45 mL; 1.25 N solution in MeOH) was added dropwise. After evaporation of the solvent compound **19 **was recovered as a brown solid (0.242 g; 40%). ^1^H-NMR (500 MHz, CD_3_OD): δ 6.28 (1H, br s, H-4'a), 5.97 (1H, br s, H-4'b), 5.35 (1H, br s, H-15a), 5.33 (1H, br s, H-15b), 5.15 (2H, m, H-8, H-14a), 5.00 (1H, br s, H-14b), 4.50 (1H, dddd, *J =* 9.0, 8.0, 2.2 and 2.2 Hz, H-3), 4.44 (1H, m, H-6), 4.30 (2H, s, H_2_-3'), 3.62 (1H, m, H-13a), 3.50–3.42 (2H, m, H-11, H-13b), 3.03–2.91 (8H, m, H-1, H-5, NH(C*H_3_*)_2_), 2.83 (1H, dd, *J =* 13.4 and 5.0 Hz, H-9a), 2.72 (1H, m, H-7), 2.33 (1H, dd, *J =* 13.4 and 7.0 Hz, H-9b), 2.24 (1H, m, H-2a), 1.75 (1H, m, H-2b); ^13^C-NMR (125 MHz, CD_3_OD): δ 177.55 (C, C-12), 166.72 (C, C-1'), 154.17 (C, C-4), 143.95 (C, C-10), 141.84 (C, C-2'), 127.82 (CH_2_, C-4'), 117.58 (CH_2_, C-14), 111.28 (CH_2_, C-15), 81.77 (CH, C-6), 77.26 (CH, C-8), 73.82 (CH, C-3), 61.98 (CH_2_, C-3'), 58.34 (CH_2_, C-13), 50.74 (CH, C-5), 49.85 (CH, C-7), 44.98 (CH, C-1), 44.77 (CH_3_, N(*C*H_3_)_2_), 42.02 (CH, C-11), 40.54 (CH, C-9), 39.51 (CH_2_, C-2).HRMS (ESI) calculated for C_21_H_30_NO_6 _[M+H]^+^, 392.2067; found 392.2062.

*Deacylcynaropicrin dimethylamine adduct* **20**. To a cold solution of **5** (0.050 g; 0.191 mmol) in absolute EtOH (5 mL), dimethylamine, (0.1 mL, 2.0 M solution in methanol) was added under argon atmosphere. The solution was stirred at 5 °C for 5 h and then the mixture was concentrated under reduced pressure. The crude residue was then purified by column chromatography on silica gel (CH_2_Cl_2_/MeOH 9:1) to afford compound **20** as a yellow oil (0.052 g; 88%). ^1^H-NMR (500 MHz, CD_3_OD): δ 5.32 (1H, br s, H-15a), 5.28 (1H, br s, H-15b), 5.05 (1H, br s, H-14a), 5.00 (1H, br s, H-14b), 4.48 (1H, dddd, *J =* 9.0, 8.0, 2.2 and 2.2 Hz, H-3), 4.16 (1H, dd, *J =* 9.8 and 9.7 Hz, H-6), 3.67 (1H, ddd, *J =* 9.0, 7.3 and 5.0 Hz, H-8), 3.02–2.80 (3H, m, H-1, H-5, H-11), 2.76 (1H, dd, *J =* 12.7 and 2.7, H-13a), 2.68 (1H, m, H-9a), 2.60 (1H, m, H-13b), 2.34 (6H, s, N(C*H_3_*)_2_), 2.26–2.14 (3H, m, H-2a, H-7, H-9b), 1.70 (1H, ddd, *J =* 12.8, 9.7 and 8.8 Hz, H-2b); ^13^C-NMR (125 MHz, CD_3_OD): δ 177.71 (C, C-12), 154.33 (C, C-4), 145.14 (C, C-10), 116.01 (CH_2_, C-14), 111.70 (CH_2_, C-15), 80.53 (CH, C-6), 74.00 (CH, C-3 and C-8), 60.81 (CH_2_, C-13), 58.34 (CH, C-7), 50.53 (CH, C-5), 46.44 (CH, C-1), 44.89 (CH_3_, N(*C*H_3_)_2_), 44.82 (CH, C-11), 43.08 (CH_2_, C-9), 39.49 (CH_2_, C-2). HRMS (ESI) calculated for C_17_H_26_NO_4_ [M+H]^+^, 308.1856; found 308.1862.

*General Procedure for Preparing Lactone Methylamines:* A solution of the appropriate enoate (1 eq.) in ethanol (0.1M) containing the required volatile amine (2.5 eq.) or non-volatile amine (0.6 eq.) and triethylamine (1.1–2.5 eq., for the appropriate hydrochloride salt) was heated at 85 °C under microwave irradiation set at 30 W for 30 min to 1 h, depending on the amine. All were prepared on a sufficiently small scale that the solutions could simply be concentrated and purified by column chromatography. The following compounds were produced this way:

*Deacylated diethylamine adduct* **21**. A mixture of **15** and its lumisantonin equivalent (~2:1 mixture, 0.128 g, 0.325 mmol) and diethylamine (68 μL, 0.663 mmol) in ethanol (3.3 mL) was stirred at room temperature for 18 h, and then concentrated to a brown oil. Column chromatography (10% ethanol/ethyl acetate as eluent) afforded an orange oil (48.9 mg, 45%); *R*_f_ 0.11 (10% ethanol/ethyl acetate); ^1^H-NMR (400 MHz, CDCl_3_) δ 4.76 (1H, br d, *J =* 9.6), 3.31–3.12 (1H, m), 2.82 (1H, dd, *J =* 3.9, 13.8), 2.68 (1H, dd, *J =* 6.0, 13.7), 2.61–2.48 (4H, m), 2.48–2.31 (5H, m), 2.09 (1H, d, *J =* 12.1), 1.97 (1H, dt, *J =* 3.5, 13.6), 1.82 (3H, br t), 1.79 (1H, td, *J =* 3.9, 13.4), 1.44–1.28 (1H, m), 0.91 (6H, t, *J =* 7.1), 0.91 (3H, s); ^13^C-NMR (101 MHz, CDCl_3_) δ 208.14, 176.47, 162.00, 161.98, 142.69, 81.45, 74.35, 52.04, 50.40, 47.18, 45.76, 45.17, 44.97, 37.15, 26.11, 21.18, 11.63, 9.41; HRMS (ESI) calculated C_19_H_30_NO_4_ 336.2175, found 336.2135 (MH^+^).

*Morpholine adduct* **23**. α-Methylenesantonin (51.7 mg, 0.213 mmol), morpholine (46.5 μL, 0.534 mmol) and ethanol (2 mL) afforded, after column chromatography (2%–4% methanol/chloroform as eluent), a yellow solid (45.7 mg, 65%); *R*_f_ 0.24 (4% methanol/chloroform). Recrystallisation yielded orange needles, mp. 165–167 °C (ethyl acetate/hexane); ^1^H-NMR (400 MHz, CDCl_3_) δ 6.71 (1H, d, *J =* 9.9), 6.26 (1H, d, *J =* 9.9), 4.82 (1H, dd, *J =* 1.3, 11.5), 3.89–3.46 (4H, m), 2.95–2.81 (1H, m), 2.71–2.57 (2H, m), 2.57–2.47 (2H, m), 2.47–2.35 (2H, m), 2.34–2.25 (1H, m), 2.12 (3H, d, *J =* 1.2), 2.05 (1H, qd, *J =* 3.5, 11.6), 1.89 (1H, ddd, *J =* 2.2, 3.6, 13.4), 1.74 (1H, ddd, *J =* 3.8, 12.9, 25.1), 1.55 (1H, td, *J =* 4.5, 13.2), 1.33 (3H, s); ^13^C-NMR (101 MHz, CDCl_3_) δ 186.22, 176.14, 154.93, 150.92, 128.52, 125.73, 81.35, 66.73, 57.70, 53.72, 51.82, 43.46, 41.12, 37.89, 25.00, 23.75, 10.80; HRMS (ESI) calculated C_19_H_26_NO_4_ 332.1862, found 332.1835 (MH^+^). The resultant dienone (0.434 g, 1.334 mmol), 5% Pd-C (0.470 g), 32% hydrochloric acid (0.5 mL) and ethanol (10 mL) afforded, after column chromatography (30%–50% acetone/hexane as eluent), an orange foam (0.209 g, 48%); *R*_f_ 0.68 (20% ethanol/ethyl acetate); ^1^H-NMR (400 MHz, CDCl_3_) δ 3.91 (1H, t, *J =* 10.6), 3.76–3.60 (4H, m), 2.82 (1H, dd, *J =* 4.3, 12.7), 2.65–2.44 (6H, m), 2.44–2.32 (3H, m), 2.19–2.08 (2H, m), 1.92–1.53 (5H, m), 1.42–1.28 (1H, m), 1.25 (3H, d, *J =* 6.6), 1.18 (3H, s); ^13^C-NMR (101MHz, CDCl_3_) δ 211.44, 177.55, 83.11, 66.85, 57.72, 53.88, 53.47, 51.11, 44.93, 43.36, 40.65, 40.28, 37.29, 36.32, 23.77, 18.39, 13.91; HRMS (ESI) calculated C_19_H_29_NaNO_4_ 358.1994, found 358.2048 (M^+^+Na^+^) and calculated C_19_H_30_NO_4_ 336.2175, found 336.2152 (MH^+^).

*Morpholine adduct* **24**. The appropriate enoate [20,21] (53.8 mg, 0.218 mmol), morpholine (17.7 μL, 0.203 mmol) and ethanol (2 mL) were mixed at 0 °C, then left to warm to room temperature for 72 h. The mixture was concentrated to afforded, after column chromatography (30% acetone/hexane as eluent), a pale yellow oil (66.0 mg, 985%); *R*_f_ 0.25 (30% acetone/hexane); ^1^H-NMR (400 MHz, CDCl_3_) δ 6.65 (1H, d, *J =* 9.9), 5.83 (1H, d, *J =* 9.9), 3.94 (1H, t, *J =* 10.7), 3.73–3.46 (4H, m), 2.76 (1H, dd, *J =* 3.6, 12.1), 2.62–2.39 (4H, m), 2.39–2.26 (2H, m), 2.14 (1H, dt, *J =* 5.7, 8.6), 2.13–2.05 (1H, m), 1.97–1.88 (1H, m), 1.80 (1H, td, *J =* 5.8, 11.5), 1.70–1.46 (3H, m), 1.29 (3H, d, *J =* 6.9), 1.11 (3H, s); ^13^C-NMR (101 MHz, CDCl_3_) δ 200.54, 177.32, 158.13, 126.55, 81.77, 66.70, 57.61, 53.77, 51.62, 51.08, 43.14, 42.09, 38.14, 37.45, 23.38, 19.18, 14.53; HRMS (ESI) calculated C_19_H_28_NO_4_ 334.2018, found 334.1974 (MH^+^).

*Tyramine adduct* **26**. The appropriate enoate [20,21] (49.7 mg, 0.202 mmol), tyramine (27.9 mg, 0.203 mmol) and ethanol (2 mL) were mixed at 0 °C, then left to warm to room temperature for 72 h. The mixture was concentrated to afforded, after column chromatography (70% acetone/hexane as eluent), a beige foam (65.5 mg, 85%); *R*_f_ 0.40 (70% acetone/hexane); ^1^H-NMR (400 MHz, CDCl_3_) δ 6.95 (2H, d, *J =* 8.4), 6.65 (2H, d, *J =* 8.4), 6.60 (1H, d, *J =* 9.9), 5.81 (1H, d, *J =* 9.9), 4.93 (1H, s), 3.91 (1H, t, *J =* 10.5), 2.91–2.72 (4H, m), 2.67 (2H, t, *J =* 7.0), 2.55–2.40 (2H, m), 1.91–1.69 (3H, m), 1.67–1.57 (1H, m), 1.57–1.36 (2H, m), 1.24 (3H, d, *J =* 6.8), 1.06 (3H, s); ^13^C-NMR (101 MHz, CDCl_3_) δ 200.76, 177.96, 158.26, 155.00, 130.33, 129.72, 126.54, 115.65, 82.15, 51.55, 51.47, 48.88, 47.26, 45.29, 42.07, 38.26, 37.22, 34.73, 29.18, 22.75, 19.16, 14.49; HRMS (ESI) calculated C_23_H_30_NO_4_ 384.2175, found 384.2130 (MH^+^).

*Tyramine adduct* **27**. Adduct **28** (0.355 g, 0.945 mmol), 5% Pd-C (0.266 g), 32% hydrochloric acid (0.5 mL) and ethanol (10 mL) afforded, after column chromatography (30%–50% acetone/hexane as eluent), a pale orange foam (0.144 g, 40%); *R*_f_ 0.44 (20% ethanol/ethyl acetate); ^1^H-NMR (400 MHz, CDCl_3_) δ 7.03 (2H, d, *J =* 8.1), 6.72 (2H, d, *J =* 8.3), 3.97 (2H, br s), 3.91 (1H, t, *J =* 10.4), 3.66 (1H, td, *J =* 0.7, 6.6), 3.03–2.80 (4H, m), 2.74 (2H, t, *J =* 6.9), 2.58–2.37 (3H, m), 1.86–1.69 (3H, m), 1.69–1.46 (3H, m), 1.33–1.23 (2H, m), 1.21 (3H, d, *J =* 6.5), 1.15 (3H, s); ^13^C-NMR (101 MHz, CDCl_3_) δ 211.56, 178.11, 154.76, 130.65, 129.75, 115.57, 83.44, 53.37, 51.48, 48.84, 47.26, 45.41, 44.85, 40.62, 39.98, 37.32, 36.39, 34.79, 23.07, 18.34, 13.81; HRMS (ESI) calculated C_23_H_31_NaNO_4_ 408.2151, found 408.2169 (M^+^+Na^+^) and calculated C_23_H_32_NO_4_ 386.2331, found 386.2299 (MH^+^).

*Tyramine adduct* **28**. α-Methylenesantonin (0.220 g, 0.910 mmol), tyramine (77.1 mg, 0.560 mmol) and ethanol (2 mL) afforded, after column chromatography (10%–20% ethanol/ethyl acetate as eluent), a yellow foam (89.7 mg, 44%); *R*_f_ 0.26 (20% ethanol/ethyl acetate); ^1^H-NMR (400 MHz, CDCl_3_) δ 7.00 (2H, d, *J =* 8.5), 6.74 (2H, d, *J =* 8.5), 6.69 (1H, d, *J =* 9.9), 6.24 (1H, d, *J =* 9.9), 4.78 (1H, dd, *J =* 1.1, 11.4), 4.72 (2H, br s), 2.98–2.88 (2H, m), 2.88–2.80 (2H, m), 2.72 (2H, t, *J =* 7.0), 2.63 (1H, dt, *J =* 6.0, 12.2), 2.08 (3H, d, *J =* 0.9), 1.98 (2H, ddd, *J =* 7.0, 15.9, 31.8), 1.88–1.76 (1H, m), 1.66 (1H, qd, *J =* 3.4, 12.8), 1.40 (1H, td, *J =* 4.2, 13.1), 1.27 (3H, s); ^13^C-NMR (101 MHz, CDCl_3_) δ 186.57, 176.76, 155.42, 155.07, 151.32, 130.17, 129.60, 128.39, 125.49, 115.52, 81.52, 58.08, 51.30, 49.55, 47.17, 45.61, 41.22, 37.51, 34.73, 24.86, 22.92, 18.20, 10.81; HRMS (ESI) calculated C_23_H_28_NO_4_ 382.2018, found 382.1970 (MH^+^).

*1-(2-Chlorophenyl)piperazine adduct* **31**. α-Methylenesantonin (49.0 mg, 0.202 mmol), 1-(2-chlorophenyl)piperazine (0.120 g, 0.516 mmol) and ethanol (2 mL) afforded, after column chromatography (2%–4% methanol/chloroform as eluent), a beige solid (65.3 mg, 73%); *R*_f_ 0.41 (4% methanol/chloroform). Recrystallisation yielded a white powder, mp >190 °C (ethyl acetate/hexane); ^1^H-NMR (400 MHz, CDCl_3_) δ 7.35 (1H, dd, *J =* 1.2, 7.9), 7.27–7.15 (1H, m), 7.06–7.01 (1H, m), 7.01–6.92 (1H, m), 6.71 (1H, d, *J =* 9.9), 6.26 (1H, d, *J =* 9.9), 4.83 (1H, d, *J =* 11.4), 2.99 (4H, s), 2.95 (1H, t, *J =* 8.4), 2.81–2.64 (4H, m), 2.60 (2H, d, *J =* 5.5), 2.33 (1H, d, *J =* 12.8), 2.14 (3H, s), 2.05 (1H, ddd, *J =* 4.2, 7.6, 17.3), 1.89 (1H, d, *J =* 13.4), 1.76 (1H, ddd, *J =* 3.7, 12.8, 25.3), 1.56 (1H, td, *J =* 4.3, 13.1), 1.34 (3H, s); ^13^C-NMR (101 MHz, CDCl_3_) δ 186.24, 176.26, 154.97, 151.03, 148.93, 130.53, 128.58, 128.49, 127.46, 125.72, 123.65, 120.19, 81.36, 57.26, 53.40, 51.87, 51.00, 43.68, 41.14, 37.92, 25.00, 23.77, 10.82; HRMS (ESI) calculated C_25_H_29_ClN_2_O_3_Na 463.1764, found 463.1759 (M^+^+Na^+^), and calculated C_25_H_30_ClN_2_O_3_ 441.1945, found 441.1870 (MH^+^).

*4-Chlorobenzylamine adduct* **32**. α-Methylenesantonin (0.218 g, 0.902 mmol), 4-chlorobenzylamine (72.0 μL, 0.589 mmol) and ethanol (2 mL) afforded, after column chromatography (10%–20% ethanol/ethyl acetate as eluent), a yellow oil (0.156 g, 69%); *R*_f_ 0.52 (20% ethanol/ethyl acetate); ^1^H-NMR (400 MHz, CDCl_3_) δ_H_ 7.28 (2H, d, *J =* 8.7), 7.24 (2H, d, *J =* 8.7), 6.71 (1H, d, *J =* 9.9), 6.24 (1H, d, *J =* 9.9), 4.83 (1H, dd, *J =* 1.4, 11.5), 3.79 (1H, d, *J =* 13.6), 3.74 (1H, d, *J =* 13.6), 2.94 (1H, dd, *J =* 4.9, 12.3), 2.82 (1H, dd, *J =* 6.1, 12.3), 2.60 (1H, ddd, *J =* 5.0, 6.0, 12.2), 2.17 (1H, dd, *J =* 3.5, 12.1), 2.14–2.06 (1H, m), 2.12 (3H, d, *J =* 1.3), 2.02–1.93 (1H, m), 1.88 (1H, ddd, *J =* 2.2, 3.6, 13.4), 1.70 (1H, ddd, *J =* 3.8, 12.9, 25.4), 1.48 (1H, td, *J =* 4.5, 13.2), 1.32 (3H, s); ^13^C-NMR (101 MHz, CDCl_3_) δ 186.15, 176.47, 154.92, 150.91, 138.22, 132.46, 129.23, 128.39, 128.31, 125.59, 81.41, 53.00, 49.16, 46.32, 46.09, 41.10, 37.58, 24.93, 23.02, 10.79; HRMS (ESI) calculated C_22_H_25_ClNO_3_ 386.1523, found 386.1474 (MH^+^).

*General Procedure Used for the Conjugate Addition of Amines to Unsaturated Isophotosantonin Derivatives:* Solutions of the enoate in absolute ethanol were dosed into 8 mL ChemSpeed reaction vessels, warmed to 30 °C and then treated with the appropriate amine in ethanol. The mixtures were agitated at 600 rpm for 18 h, concentrated to gums and purified by column chromatography. In this fashion the following were prepared:

*Morpholine adducts* **22*** and* **25**. A mixture of **15** and its lumisantonin equivalent (~2:1 mixture, 0.100 g, 0.331 mmol), morpholine (57.3 μL, 0.657 mmol) and ethanol (5 mL) were treated as per the general procedure. The resultant orange gum afforded, after column chromatography (50% ethyl acetate/hexane – ethyl acetate as eluent), **22** (50.8mg, 39%); *R*_f_ 0.45 (ethyl acetate); ^1^H-NMR (400 MHz, CDCl_3_) δ 4.77 (1H, d, *J =* 10.5), 4.12 (1H, dq, *J =* 2.2, 6.3), 3.72–3.51 (5H, m), 2.79 (1H, dd, *J =* 4.1, 13.3), 2.68–2.59 (1H, m), 2.56 (1H, dd, *J =* 4.5, 13.6), 2.52–2.29 (10H, m), 2.09 (1H, dt, *J =* 3.6, 13.6), 1.94 (3H, s), 1.83 (3H, dd, *J =* 1.6, 2.2), 1.41 (1H, tdd, *J =* 3.4, 10.8, 14.0), 1.01 (3H, s); ^13^C-NMR (101 MHz, CDCl_3_) δ 206.91, 175.75, 170.33, 160.68, 143.13, 85.51, 81.19, 66.61, 57.11, 53.97, 47.13, 45.81, 43.80, 37.89, 36.70, 25.41, 22.23, 19.97, 9.45; HRMS (ESI) calculated C_21_H_30_NO_6_ 392.2073, found 392.2047 (MH^+^). Also isolated was **25 **slightly contaminated with the former product, as an orange gum (34.4mg, 32%); *R*_f_ 0.33 (ethyl acetate). An analytical sample was purified by preparative TLC (ethyl acetate): ^1^H-NMR (400MHz, CDCl_3_) δ 6.70 (1H, d, *J =* 9.9), 6.27 (1H, d, *J =* 9.9), 4.80 (1H, dq, *J =* 1.3, 11.5), 3.80–3.50 (4H, m), 2.98–2.75 (1H, m), 2.70–2.57 (2H, m), 2.57–2.48 (2H, m), 2.48–2.36 (2H, m), 2.36–2.25 (1H, m), 2.13 (3H, d, *J =* 1.4), 2.05 (1H, qd, *J =* 3.5, 11.7), 1.88 (1H, ddd, *J =* 2.2, 3.6, 13.4), 1.72 (1H, ddd, *J =* 3.8, 12.9, 25.0), 1.55 (1H, td, *J =* 4.5, 13.2), 1.33 (3H, s); ^13^C-NMR (101 MHz, CDCl_3_) δ 186.29, 186.28, 176.15, 154.88, 150.82, 128.75, 125.89, 81.45, 66.82, 57.79, 53.82, 51.90, 43.60, 41.17, 37.99, 25.10, 23.88, 10.88; HRMS (ESI) calculated C_19_H_26_NO_4_ 332.1862, found 332.1836 (MH^+^).

*Tyramine adducts* **29*** and* **30**. A mixture of **15** and its lumisantonin equivalent (~2:1 mixture, 0.101 g, 0.331 mmol), tyramine (90.2 mg, 0.657 mmol) and ethanol (5 mL) were treated as per the general procedure. The resultant brown gum afforded, after column chromatography (50% ethyl acetate:hexane – ethyl acetate as eluent), **29** (60.3 mg, 41.3%); *R*_f_ 0.25 (ethyl acetate); ^1^H-NMR (400 MHz, CDCl_3_) δ 7.13–6.98 (2H, m), 6.84–6.68 (2H, m), 4.98 (1H, d, *J =* 10.7), 4.16 (1H, br m), 3.00–2.80 (4H, m), 2.76 (2H, d, *J =* 6.7), 2.65–2.46 (4H, m), 2.46–2.37 (1H, m), 2.18 (1H, dd, *J =* 3.4, 13.5), 2.09 (1H, s), 2.03 (3H, s), 1.89 (3H, d, *J =* 1.5), 1.53 (1H, ddd, *J =* 3.2, 10.5, 14.3), 1.37–1.19 (1H, m), 1.11 (3H, s); ^13^C-NMR (101 MHz, CD_3_OD) δ 208.56, 177.44, 171.36, 162.40, 155.73, 143.28, 130.61, 130.15, 130.03, 116.02, 115.80, 86.24, 82.10, 51.93, 47.90, 47.30, 46.40, 44.54, 38.21, 37.29, 35.31, 25.52, 22.54, 20.16, 9.64; HRMS (ESI) calculated C_25_H_32_NO_6_ 442.2230, found 442.2229 (MH^+^). Also isolated was **30** slightly contaminated with the former product, as an orange gum (21.9 mg, 17.4%); *R*_f_ 0.14 (ethyl acetate). An analytical sample was purified by preparative TLC (ethyl acetate): ^1^H-NMR (400 MHz, CD_3_OD) δ_H_ 7.04 (2H, d, *J =* 8.5), 6.73 (2H, d, *J =* 8.5), 6.69 (1H, d, *J =* 9.9), 6.26 (1H, d, *J =* 9.9), 4.79 (1H, dd, *J =* 1.4, 11.4), 2.99 (1H, dd, *J =* 5.0, 12.4), 2.84 (4H, m), 2.72 (2H, t, *J =* 6.8), 2.63–2.54 (1H, m), 2.11 (3H, d, *J =* 1.3), 2.10–2.03 (1H, m), 2.03–1.94 (2H, m), 1.88 (1H, ddd, *J =* 2.1, 3.7, 13.4), 1.74 (1H, ddd, *J =* 3.9, 13.1, 25.8), 1.50 (1H, td, *J =* 4.1, 13.1), 1.33 (3H, s); ^13^C-NMR (101 MHz, CD_3_OD) δ 186.43, 176.61, 154.93, 154.15, 150.86, 131.40, 129.73, 128.72, 125.84, 115.34, 81.57, 51.51, 49.41, 47.11, 46.15, 41.20, 37.76, 35.09, 25.04, 23.23, 10.90; HRMS (ESI) calculated C_23_H_28_NO_4_ 382.2018, found 382.1986 (MH^+^).

*2-(4-Chlorophenyl)ethylamine adducts* **33** *and* **34**. A mixture of **15** and its lumisantonin equivalent (~2:1 mixture, 0.114 g, 0.376 mmol), 2-(4-chlorophenyl)ethylamine (91.4 μL, 0.657 mmol) and ethanol (5 mL) were treated as per the general procedure. The resultant orange gum afforded, after column chromatography (50% ethyl acetate/hexane – ethyl acetate as eluent), **33** (60.8 mg, 35%); *R*_f_ 0.27 (ethyl acetate); ^1^H-NMR (400 MHz, CDCl_3_) δ _H_ 7.19 (2H, d, *J =* 8.4), 7.07 (2H, d, *J =* 8.5), 4.76 (1H, d, *J =* 10.5), 4.10–4.04 (1H, m), 2.93 (1H, dd, *J =* 3.9, 12.4), 2.87–2.72 (3H, m), 2.69 (2H, t, *J =* 6.9), 2.55–2.36 (4H, m), 2.36–2.27 (1H, m), 2.11–1.98 (2H, m), 1.94 (3H, s), 1.81 (3H, dd, *J =* 1.6, 2.1), 1.37 (1H, tdd, *J =* 3.4, 10.7, 14.1), 1.00 (3H, s); ^13^C-NMR (101 MHz, CDCl_3_) δ 206.90, 176.18, 170.23, 160.70, 143.02, 138.14, 131.82, 129.98, 128.44, 85.40, 81.46, 51.29, 47.15, 46.62, 46.50, 43.48, 37.71, 36.71, 35.48, 25.29, 22.21, 19.89, 9.41; HRMS (ESI) calculated C_25_H_31_ClNO_5_ 460.1891, found 460.1866 (MH^+^). Also isolated was **34 **slightly contaminated with the former product, as an orange gum (28.6mg, 19.0%); *R*_f_ 0.15 (ethyl acetate). An analytical sample was purified by preparative TLC (ethyl acetate): ^1^H-NMR (400 MHz, CDCl_3_) δ 7.25 (2H, d, *J =* 8.5), 7.13 (2H, d, *J =* 8.5), 6.70 (1H, d, *J =* 9.9), 6.27 (1H, d, *J =* 9.9), 4.80 (1H, dd, *J =* 1.4, 11.4), 2.96 (1H, dd, *J =* 5.3, 12.3), 2.90 (1H, dd, *J =* 5.8, 11.9), 2.89–2.80 (3H, m), 2.80–2.72 (2H, m), 2.62–2.52 (1H, m), 2.13 (3H, d, *J =* 1.3), 2.02 (1H, ddd, *J =* 3.7, 12.1, 23.8), 2.02–1.94 (1H, m), 1.86 (1H, ddd, *J =* 2.2, 3.7, 13.5), 1.68 (1H, ddd, *J =* 3.8, 12.9, 25.3), 1.47 (1H, td, *J =* 4.5, 13.3), 1.32 (3H, s); ^13^C-NMR (101 MHz, CDCl_3_) δ 186.28, 176.57, 154.83, 150.72, 138.21, 131.91, 130.01, 128.74, 128.50, 125.88, 81.56, 51.28, 49.51, 47.30, 46.35, 41.18, 37.72, 35.63, 25.12, 23.30, 10.93; HRMS (ESI) calculated C_23_H_27_ClNO_3_ 400.1679, found 400.1645 (MH^+^).

### 3.4. Sample Preparation for Biological Testing

Compounds were dissolved in DMSO (10 mg/mL) and stored at −20 °C until testing. Fresh dilutions in medium were prepared for each bioassay. DMSO concentration in the assay did not exceed 1%. All assays were performed in at least three independent experiments. The purity of all compounds was >95% if not stated otherwise. 

### 3.5. Trypanosoma Brucei Rhodesiense (STIB 900 strain) Bioassay

Evaluation of *in vitro* antiprotozoal activity against *T. b. rhodesiense* was done using the Alamar Blue assay to determine IC_50_s as previously described [24]. Serial threefold dilution were prepared in 96-well micro titre plates, and 4000 *T. b. rhodesiense* STIB 900 bloodstream forms in 50 μL were added to each well except for the negative controls. Melarsoprol (Arsobal^®^, purity > 95%, Sanofi-Aventis, Meyrin, Switzerland) was used as reference drugs. After 70 h of incubation 10 μL of Alamar blue marker (12.5 mg resazurin (Sigma-Aldrich, Buchs, Switzerland) dissolved in 100 mL of distilled water) was added, and colour change was developed for 2 to 6 h. A Spectramax Gemini XS micro plate fluorescence reader (Molecular Devices Cooperation, Sunnyvale, CA, USA) with an excitation wavelength of 536 nm and an emission wavelength of 588 nm was used to read the plates. The IC_50_ values were calculated from the sigmoidal growth inhibition curves using Softmax Pro software (Molecular Devices). 

### 3.6. Rat Myoblast Cell L6-Cytotoxicity Assay

The cytotoxicity assay was performed with rat skeletal myoblasts (L6-cells) seeded in 100 μL RPMI 1640 in 96-well micro titre plates, using the Alamar Blue assay described above. After 24 h the medium was removed and replaced by 100 μL of fresh RPMI 1640 with serial threefold drug dilution. Podophyllotoxin (purity > 95%, Sigma-Aldrich) was used as a reference drug. After 70 h of incubation under a humidified 5% CO_2_ atmosphere, 10 μL of the Alamar blue marker (see above) was added to all wells. The plates were incubated for an additional 2 h. A Spectramax Gemini XS micro plate fluorescence reader (Molecular Devices) was used to read the plates using an excitation wavelength of 536 nm and an emission wavelength of 588 nm. The IC_50_s were calculated from the sigmoidal growth inhibition curves using Softmax Pro software (Molecular Devices).

### 3.7. Acute Mouse Sleeping Sickness Model

This model mimics the first stage of the human African trypanosomiasis. Adult female NMRI mice were purchased from Janvier (St. Berthevin, France). They weighed between 20 and 25 g at the beginning of the study and were kept under standard conditions at 22 °C and 60%–70% humidity in macrolon type III cages with food pellets and water *ad libitum*. All protocols and procedures used in this study were reviewed and approved by the local veterinary authorities of the Canton Basel-Stadt, Switzerland (authorization N 739; 11.12.2009). The samples were first dissolved in 100% DMSO, followed by addition of distilled H_2_O to a final DMSO concentration of 10%. For determination of the *in vivo* antitrypanosomal activity, mice were infected intraperitoneally with 1 × 10^4^ STIB900 bloodstream forms. Experimental groups of four mice were treated orally once a day on four consecutive days from day 3 to day 6 post infection. A control group of four mice was infected, but remained untreated. The determination of the parasitaemia was done on day 7 post infection. 6 μL of tail blood were diluted in 24 μL sodium citrate (3.2%), whereby the first μL was discarded to obtain circulating blood. Five μL of this mixture were transferred to a glass slide and covered with an 18 × 18 mm cover slide. The sample was examined under a light microscope (200-fold magnification) and parasites were counted in 3 of the 16 squares of the grid.

## 4. Conclusions

The conclusions are that the α-methylene-γ-lactone is necessary for both antitrypanosomal effects and cytotoxicity of these sesquiterpenes. Antitrypanosomal selectivity is facilitated by 2-(hydroxyl-methyl)acrylate or 3,4-dihydroxy-2-methylenebutylate side chains, and by the presence of cyclopentenone rings. Semi-synthetic STL amines with improved activity over the native STLs were those with morpholino and dimethylamino groups. The dimethylamino analogue of cynaropicrin was prepared and tested orally in the *T. b. rhodesiense* acute mouse model, where it showed reduced toxicity over cynaropicrin, but also reduced antitrypanosomal effects.
